# Prevalence of latent tuberculosis infection among foreign students in Lübeck, Germany tested with QuantiFERON-TB Gold In-Tube and QuantiFERON-TB Gold Plus

**DOI:** 10.1186/s12995-017-0159-4

**Published:** 2017-05-25

**Authors:** Elia Noemi Gallegos Morales, Johannes Knierer, Anja Schablon, Albert Nienhaus, Jan Felix Kersten

**Affiliations:** 1 Center of Excellence for Health Services Research in Nursing (CVcare), University Medical Center Hamburg-Eppendorf (UKE), Hamburg, Germany; 2 Institute for Statutory Accident Insurance and Prevention in the Health and Welfare Services (BGW), Hamburg, Germany

**Keywords:** Foreign students, Latent tuberculosis infection, Risk factors, QuantiFERON-TB Gold Plus

## Abstract

**Background:**

The tuberculosis (TB) incidence rate in foreign-born individuals has been increasing in Germany in recent years. Foreign students may be an important source of latent tuberculosis infection (LTBI) in low-incidence countries. In Germany, there are no guidelines for LTBI screening of foreign students. The aim of the study was to estimate LTBI prevalence and evaluate associated risk factors among foreign students in Germany. The second purpose of our study was to compare the results of the new generation of QuantiFERON-TB Gold Plus (QFT-Plus) to those of its predecessor QuantiFERON-TB Gold In-Tube (QFT-GIT).

**Methods:**

This cross**-**sectional study was conducted between February 2016 and March 2016. Foreign students and young professionals attending the university and higher education institutes in Lübeck, Germany were tested with QFT-Plus and QFT-GIT. Participants filled out a questionnaire for the purpose of LTBI risk assessment and analysis. Variables associated with a positive test result were analyzed using logistic regression.

**Results:**

One hundred thirty four students participated in the study. The overall prevalence as regards positive results from both tests, QFT-Plus and QFT-GIT, was 9.7%, and the prevalence of positive QFT-Plus results was 8.2%. The main independent variables associated with a positive QFT-Plus result were a) being born in a high-incidence country (OR = 6.7, 95% CI: 1.3–34.3) and b) previous contact with a person with active TB (OR = 4.5, 95% CI: 1.1–18.3). Higher age (OR = 2.8, 95% CI: 0.7–11.3) and male gender (OR = 1.6, 95% CI: 0.4–6.7) showed a tendency toward positive QFT-Plus results but this was not statistically significant. Agreement between QFT-Plus and QFT-GIT results was κ = 0.85, *p* < 0.001.

**Conclusions:**

The LTBI prevalence among foreign students was about 10%. We recommend implementing a policy whereby all foreign students are screened by means of a questionnaire about LTBI risk factors, so that only students with present risk factors are tested for LTBI. The agreement between the new QFT-Plus and the QFT-GIT (κ = 0.85) was good. QFT-Plus might be used in the same format as its predecessor.

## Background

The downward trend of tuberculosis (TB) incidence in Germany has come to an end. TB incidence in Germany had decreased over the last decades, reaching its lowest level in 2012, with 4217 reported TB cases (TB incidence of 5.2 new TB cases a year per 100,000 inhabitants (hereinafter 5.2:100,000 for short)). Since then, TB incidence has been increasing and reached 5865 reported cases of active TB in 2015, 1648 more TB cases than in 2012. Nevertheless, Germany is still a low TB incidence country with a TB incidence of 7.3:100,000 in 2015 [1].

The overall TB incidence in Germany increased in recent years, mainly due to the increased number of TB cases in foreign-born people residing in Germany. All TB cases considered, the proportion of foreign-born people increased from 43.1% in 2007 to 72.1% in 2015. TB incidence in foreign-born subjects (50.3:100,000) was 20 times higher than the incidence in the native population (2.5:100,000) in 2015. (TB incidence rate 2007 in foreign-born residents: 22.8:100,000 and in native population: 4.3:100,000). Among foreign-born subjects with active TB, the peak age for TB was between 15 and 19 years [1].

Diel et al. [2] explained the considerable increase of TB incidence in Germany during recent years by the higher numbers of asylum seekers entering the country since 2014. However, not only has the number of asylum seekers increased in recent years, but also the number of foreign students attending universities and higher education institutions in Germany. The German Federal Statistical Office revealed in a press release that in the 2014/2015 winter term 236,000 foreign students were registered at German higher education institutions and universities. The number of foreign students has been increasing for the past ten years (2004/2005 winter term: 187,000 foreign students) [3].

The main goal of the World Health Organization (WHO) End TB Strategy is to end the global TB epidemic by 2035. One of the three pillars of this strategy is TB prevention. An important component in TB prevention is the management of latent tuberculosis infection (LTBI) in countries with a lower TB incidence (<100:100,000) by preventive treatment of persons at risk. A large proportion of TB cases in these countries are due to LTBI reactivation. Preventive therapy could reduce the risk of developing active TB by 60–90% [4, 5].

In the United States, the American College Health Association (ACHA) has developed guidelines for incoming students that recommend screening and targeted testing of college and university students to control and prevent infection by *Mycobacterium tuberculosis* [6]. Collins et al. found that in the U.S., students accounted for 30% of the TB cases among temporary visa holders [7]. This suggests that students might make up a significant proportion of residents with TB in the U.S. Ogiwara et al. proposed that early LTBI screening among foreign students could reduce the spread of TB [8]. Ricks et al. implied that four out of five active TB cases among foreign-born persons in the U.S. are attributed to the reactivation of LTBI. Preventing LTBI reactivation represents a big challenge to TB elimination [9].

Currently, no policy exists that recommends systematic testing for LTBI among foreign students prior to or during the process of entering Germany. To our knowledge, the prevalence and risk factors for LTBI among students and young professionals recently migrated to Germany has not yet been studied.

The WHO recommends using either tuberculin skin test (TST) or interferon-gamma release assay (IGRA) to test for LTBI in high-income and upper middle-income countries with an estimated TB incidence of ≤100:100,000 [10]. Until recently two IGRAs were commercially available, T-SPOT.TB (Oxford Immunotec, Abingdon, UK) and QuantiFERON-TB Gold In-Tube (QFT-GIT) (Qiagen, Hilden, Germany). In 2015 Qiagen released the QuantiFERON-TB Gold Plus (QFT-Plus), which according to the manufacturer offers a higher sensitivity and specificity [11].

The aim of this cross-sectional study is to estimate the prevalence and the associated risk factors of a positive IGRA as a marker for LTBI among students and young professionals with a migration background in Lübeck, Germany. The second purpose of our study is to compare the results of the new generation IGRA, QFT-Plus, to those of its predecessor, QFT-GIT.

## Methods

### Study design, setting and subjects

This cross-sectional study was conducted between February 2016 and March 2016. The target population for this cross-sectional study was foreign students and young professionals attending courses offered by the university and two higher education institutions in Lübeck, Germany. Students were recruited by word-of-mouth advertising, flyers on notice boards and e-mails sent from the international office of each institution. Students attending universities other than those in Lübeck were not directly recruited, but their participation in this study was allowed.

Students participated in this study on a voluntary basis and their contribution was remunerated. The inclusion criteria were: age ≥ 18 years, being a student or a young professional attending a German university/higher education institution, having signed the consent form and being born in or being a national of a country with a TB incidence ≥10:100,000 according to the WHO [12]. Participants without a migration background fulfilling the nationality criteria only because of their parents’ nationality were excluded. Students fulfilling the inclusion criteria attended an appointment at the University of Lübeck where data was collected and blood was drawn. A Palestinian participant was included in the study, although there is no TB incidence data for that country according to the WHO.

We declared as a high-incidence country all those countries with a TB incidence of ≥125:100,000. The participants’ nationality was classified by WHO regions.

### Data collection

After signing the consent form, participants filled out a standard questionnaire asking about risk factors. The questionnaire included questions about socio-demographic aspects (gender, age, country of birth, nationality, time living in Germany), subject studied, previous contact with TB, TB in the participants’ own history, previous TB test results (IGRA and TST), BCG (*Bacille Calmette-Guérin*) vaccination, previous chest X-rays and other diseases or medications that compromise the immune system.

### Test method: IGRA

The participants’ blood samples were tested by two IGRA methods: QFT-GIT and QFT-Plus (both: Qiagen, Hilden, Germany).

Students were tested at the University of Lübeck, where rooms were booked for study purposes only. Blood sampling was performed by two study physicians using lithium heparin tubes. The blood samples were stored in a room temperature box and transported to the laboratory within max. 8 h. In the laboratory, medical lab technicians decanted an exact amount of blood from the heparin tubes into the QFT-GIT and the QFT-Plus tubes. The samples were incubated for at least 16 h and Interferon-gamma (IFN-γ) ELISA (Enzyme-linked Immunosorbent Assay) was performed directly afterwards.

Results were considered positive if the IFN-γ value was ≥0.35 IU/ml after correction for negative control in any of the two tests. A result was considered negative if the IFN-γ value was <0.35 IU/ml and if the mitogen (positive response control) was ≥0.5 IU/ml. Since the QFT-Plus has two tubes, TB-antigen 1 and TB-antigen 2, the maximum of the two was taken as the result, as per the manufacturer’s suggestion [13].

### Management of IGRA-positive participants

All participants were informed about their results. Participants with a positive test result in at least one of the two IGRAs were advised by the study physicians to repeat the tests in order to verify the test result (costs for follow-up tests were covered by the study). If the test kept being positive, participants were advised to take a chest X-ray in order to rule out active TB. Repetition of the IGRA was performed one week after the first positive test was performed in the scope of a four week variability study. Results of this study are reported else were [14]. Participants who had had prior contact with a person with active TB during the last 2 years were offered specialist advice. Participants with a positive test result were informed about the symptoms of active TB and about risk factors leading to LTBI reactivation.

### Data analysis

Categorical variables were expressed as counts and corresponding rates and continuous variables were expressed as means with standard deviation (SD) or with a 95% confidence interval (95%-CI). Associations between the IGRA result and categorical variables were investigated using chi-squared tests. The variables (gender, age, country of birth and previous TB contact) that were associated univariate with the QFT-Plus result in the chi-squared test were included in a logistic regression model with stepwise backward selection. Agreement between the QFT-GIT and QFT-Plus was assessed using the Cohen’s kappa coefficient. All data was analyzed with SPSS Version 22 (IBM Corp., Armonk, NY). Two-tailed *P*-value of <0.05 was considered statistically significant.

## Results

The study involved 134 foreign students and young professionals. Four out of 134 participants were enrolled in other universities than those in Lübeck. Questionnaires were filled in completely, there was no missing data. The study population is described in Table 1. In our sample the mean age was 25.1 years (SD 3.8) with an age range from 19 to 39 years. Gender was almost equally distributed, with a slightly higher proportion of female participants (53.7%). Participants migrated to Germany at ages ranging from 17 months (1.4 years) to 36 years. The mean age in which students migrated was 20.9 years.

**Table 1:** Description of the study population and QFT-Plus results by putative risk factors

Variable	N (Col-%)	QFT-Plus positive	QFT-Plus negative	*P*-Value
		N (Row-%)	N (Row-%)	
Gender				0.345
Female	72 (53.7)	4 (5.6)	68 (94.4)	
Male	62 (46.3)	7 (11.3)	55 (88.7)	
Age				0.050
19–25 years	87 (64.9)	4 (4.6)	83 (95.4)	
26–39 years	47 (35.1)	7 (14.9)	40 (85.1)	
Country of birth classified by TB-Incidence				0.002
< 125:100,000	86 (64.2)	2 (2.3)	84 (97.7)	
≥ 125:100,000	48 (35.8)	9 (18.8)	39 (81.3)	
Nationality classified by WHO regions				<0.001
Africa	15 (11.2)	4 (26.7)	11 (73.3)	
The Americas	16 (11.9)	0 (0)	16 (100)	
South-East Asia	20 (14.9)	3 (15.0)	17 (85.0)	
Europe	49 (36.6)	0 (0)	49 (100)	
Eastern Mediterranean	20 (14.9)	0 (0)	20 (100)	
Western Pacific	14 (10.4)	4 (28.6)	10 (71.4)	
Time living in Germany				0.808
< 2 years	53 (39.6)	4 (7.5)	49 (92.5)	
2–10 years	71 (53.0)	7 (9.9)	64 (90.1)	
> 10 years	10 (7.5)	0 (0)	10 (100)	
Previous TB contact				0.007
Yes	35 (26.1)	7 (20.0)	28 (80.0)	
No	99 (73.9)	4 (4.0)	95 (96.0)	
Type of TB contact				0.009
Relatives	9 (25.7)	5 (55.6)	4 (44.4)	
Friends/acquaintances	7 (20.0)	1 (14.3)	6 (85.7)	
Professional	14 (40.0)	0 (0)	14 (100)	
Other type	5 (14.3)	1 (20.0)	4 (80.0)	
TB in own history				0.082
Yes	1 (0.7)	1 (100)	0 (0)	
No	133 (99.3)	10 (7.5)	123 (92.5)	
BCG vaccination				0.064*
Yes	66 (49.3)	2 (3.0)	64 (97.0)	
No	18 (13.4)	3 (16.7)	15 (83.3)	
Unknown	50 (37.3)	6 (12.0)	44 (88.0)	
Previous TST				0.306
Positive	11 (8.2)	2 (18.2)	9 (81.8)	
Negative	18 (13.4)	0 (0)	18 (100)	
Unknown	6 (4.5)	0 (0)	6 (100)	
No TST	99 (73.9)	9 (9.1)	90 (90.9)	
Study subject				0.350
Medicine	47 (35.1)	5 (10.6)	42 (89.4)	
Engineering	49 (36.6)	3 (6.1)	46 (93.9)	
Natural sciences	21 (15.7)	3 (14.3)	18 (85.7)	
Other subject	17 (12.7)	0 (0)	17 (100)	

*For BCG vaccination variable, *P*-Value was calculated between participants whose BCG status was known

A total of 11 positive QFT-Plus results (8.2%) were observed. When both tests (QFT-GIT and QFT-Plus) are taken into account the number of participants with a positive test result in either QFT increases to a total of 13 (9.7%).

As the agreement between QFT-Plus and QFT-GIT was high, presentation of IGRA results by putative risk factors was limited to the QFT-Plus (Table 1). The prevalence of positive QFT-Plus results was higher among male participants (11.3% versus 5.6%, *p* = 0.34) and among participants born in a TB high-incidence country (18.8% versus 2.3%). The participants with a positive QFT-Plus were nationals of three WHO regions, Africa, South-East Asia and the Western Pacific, each region accounting for about one-third of positive results. None of the participants from Europe was QFT-Plus positive. Subjects reporting a previous contact with a person with active TB were more often QFT-Plus positive than those with no known contact (20% versus 4%). In most cases (5 of 7; 71%) the contact person was a relative. One participant had suffered from active TB two years before participating in the study.

Out of the 13 participants with positive IGRA results in at least one of the two IGRA test methods, 11 participants consented to retesting. Upon second testing, positivity was confirmed in at least one of the two IGRA test methods in all cases. None of the chest X-rays of participants with a positive IGRA showed signs of ongoing active TB. Despite the study physicians’ advice, four of the participants refused to take a chest X-ray. As none of the participants showed clinical signs of ongoing active TB, we assumed that no participant was suffering from active TB.

**Fig. 1:**
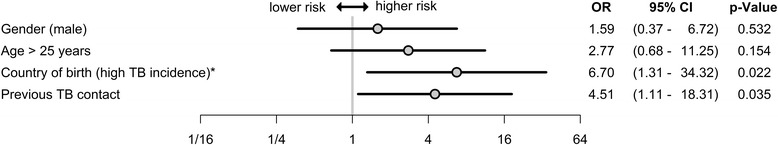
Variables associated with positive QFT-Plus. The variables were analyzed using logistic regression models to identify independent variables associated with a positive QFT-Plus result. * Countries with an annual TB incidence of ≥125:100,000

As shown in Fig. 1, we found that statistically significant risk factors for having a positive QFT-Plus as an LTBI marker were: being born in a high-incidence country (OR = 6.7, 95% CI: 1.3–34.3) and having had previous contact with a person with active TB (OR = 4.5, 95% CI: 1.1–18.3). A higher age (OR = 2.8, 95% CI: 0.7–11.3) and male gender (OR = 1.6, 95% CI: 0.4–6.7) showed a tendency to have a higher risk of acquiring LTBI, however these factors showed no statistical significance.

**Fig. 2:**
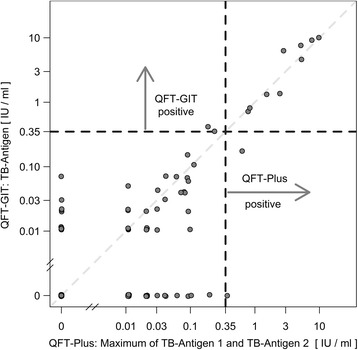
Concordance between QFT-Plus and QFT-GIT. For QFT-Plus the highest IFN-γ value of antigen tubes TB-1 and TB-2 was taken as the result

The participants were tested simultaneously with QFT-Plus and QFT-GIT. Fig. 2 shows the concordance of these two test methods. Nine participants showed positive results in both tests. Two participants were only positive in the QFT-Plus test while another two participants, who were also born in a TB high-incidence country, had a positive QFT-GIT result (Fig. 2). The concordance between QFT-Plus and QFT-GIT was estimated as κ = 0.85 (*p* < 0.001).

## Discussion

This cross-sectional study is to our knowledge the first to determine the prevalence of LTBI among foreign students and young professionals in Germany. We found an overall prevalence of positive QFT-Plus of 8.2%, which is considerably higher than with students in low-incidence countries. In Italy, Durando et al. conducted two studies in which LTBI prevalence among medical students was 0.1% (test method IGRA) in 2015 [15] and among healthcare students 0.5% (test method IGRA) in 2013 [16]. Schablon et al. found an LTBI prevalence of 2.1% (test method IGRA) among nursing students working in Germany [17]. In South Korea LTBI prevalence according to Jung et al. was 5.2% (test method IGRA) among medical students [18]. Ogiwara et al. showed that in different years the LTBI prevalence (test method IGRA) among Japanese healthcare students ranged from 0.7 to 1.0% while among foreign students the LTBI prevalence was 4.3 to 8.6% [8].

Four studies among high TB burden countries were conducted. In students from Rio de Janeiro, Brazil, the LTBI prevalence was 6.9% (test method TST) according to Teixeira et al. [19]. Du et al. showed that LTBI prevalence among Chinese students was 40.7% (test method IGRA) [20]. LTBI prevalence among Ugandan medical students was 44.8% and 35.2% among veterinary students according to Mugerwa et al. [21] and 45.1% according to Lou et al. [22], which is considerably higher than our numbers. Nevertheless, in the Ugandan studies TST was used as a test method for investigating LTBI prevalence. This might have led to an overestimation of LTBI prevalence due to cross reactivity of TST with the BCG vaccination [20, 23]. The latter three studies conducted in Uganda and in China imply that the LTBI prevalence in students from high TB burden countries is much higher than in other countries (e.g. Italy, Germany, etc.).

As there is no data available on LTBI prevalence among students from Lübeck no comparison is possible. However the TB incidence of the district Lübeck was 4.7:100,000 in 2015 and therefore lower than the TB incidence in Germany (7.3:100,000). We assume that LTBI prevalence is lower in students from Lübeck than in foreign students [1].

Our risk assessment analysis demonstrated that being born in a country with a high TB incidence (TB incidence ≥125:100,000) is a major risk factor for IGRA positivity and therefore for an LTBI among foreign students. This association has also been demonstrated in Italy [16] and in Japan [8], though the definition of a high-incidence country in these two studies was a TB incidence of ≥20:100,000. As a second risk factor for IGRA positivity we identified previous contact with a person infected with active TB. This was also demonstrated in students from Uganda and Italy [15, 21, 22].

Fournier et al. described in their study that family TB contact leads to a higher LTBI rate than professional contact in low-incidence and in high-incidence countries [24]. This corresponds to our results, since in our population the majority of participants with a positive QFT-Plus test that reported previous contact with TB had had contact with an infected family member.

In other studies male gender [19, 21, 22] and a higher age [22] were a significant predictor for an LTBI. Our data shows the same tendency for a higher risk for these two variables. However, due to the small number of participants and therefore limited power it was not statistically significant.

Our secondary assessment showed that the agreement between the new QFT-Plus and the QFT-GIT (κ = 0.85) was far greater than that between TST and QFT-GIT found by Jung et al. (κ = 0.34), which is poor [18]. This agreement is expected because QFT-GIT and QFT-Plus are based on the same testing methods and principles.

Studies have demonstrated that active TB might be attributed to reactivation of LTBI [9, 25]. This reactivation was shown to be higher among foreign-born than among natives in the U.S. [26]. Ricks et al. showed that the odds of TB reactivation were higher among foreign-born people from countries with a TB incidence of ≥150:100,000 [9]. Diel et al. showed that in Germany the majority (58.1%) of all immigrants developed the disease within the first 5 years [25]. Since students stay in Germany for several years, LTBI reactivation might occur during their residence in Germany. In a review, Ai et al. stated that the LTBI reactivation rate is especially high for persons who had been recently infected (15 times greater within the first 2 years after infection) [27].

As LTBI is an important source of active TB infection in low-incidence countries, the WHO has released guidelines specifically for the management of LTBI. The WHO found evidence for increased prevalence of LTBI, higher risk of progression (LTBI reactivation) and higher incidence of active TB among immigrants from high TB burden countries. Therefore they recommend countries with a TB incidence of <100:100,000 to consider systematic testing and treatment of LTBI for immigrants from high TB burden countries [10] (currently: Afghanistan, Bangladesh, Brazil, Cambodia, China, DR Congo, Ethiopia, India, Indonesia, Kenya, Mozambique, Myanmar, Nigeria, Pakistan, Philippines, the Russian Federation, South Africa, Thailand, Uganda, UR Tanzania, Vietnam and Zimbabwe [5]).

Although some countries such as the UK and Canada have already implemented LTBI screening policies for incoming immigrants [28–30], there are no similar regulations in Germany. Diel et al. suggested that public health policies should focus on TB prevention with LTBI testing of all immigrants applying for a resident visa in Germany [25]. In the U.S., the ACHA guidelines recommend screening all incoming students for TB by using a questionnaire inquiring about six different risk factors for TB infection. Students showing at least one risk factor (e.g. students who were born in or had visited a country with a TB incidence of ≥20:100,000, exposure to TB, including professional contact) should be tested with TST or IGRA [6]. We suggest creating a similar policy to the ACHA Guidelines with targeted testing. Since everyone has a different risk profile for LTBI, untargeted systematic testing would include testing of low-risk persons which may lead to a reduction in the positive predictive value of IGRAs [31]. In order to preserve the positive predictive value of IGRAs, only risk groups should be undergoing LTBI testing with IGRAs.

According to our findings we recommend that foreign students should be screened with a questionnaire inquiring about risk factors before the decision to test is taken. Those who are screened as having had prior contact with a person with active TB or who come from a high-incidence country (≥125:100,000 inhabitants) or a high TB burden country should be tested for LTBI. We agree with the WHO recommendation to ask students about active TB symptoms before LTBI testing. If someone suffers from these symptoms, active TB should be investigated [10].

Countries with a TB incidence of <100:100,000 should test with either TST or IGRA [10]. It is known that the TST shows cross reactivity with the BCG vaccination [20, 23] and the majority of TB high incidence countries carry out BCG vaccination [32]. Therefore IGRAs are more useful for LTBI evaluation in BCG vaccinated individuals [33] and should be preferred when testing migrants in Germany with positive risk factors.

Pareek et al. showed in a review that successful LTBI screening involves key steps e.g. accurate identification of migrants, appropriate screening and initiation as well as completion of chemotherapy [28]. LTBI reactivation and thus the spread of the disease can be averted by means of preventive chemotherapy [10]. Nevertheless, Diel et al. in 2004 note that preventive therapy might not be useful in some immigrant groups in Germany, such as those originating from Eastern Europe, since people from those countries are more likely to be infected with resistant *Mycobacterium tuberculosis* [25].

### Strengths and limitations

Participants in this study with a positive QFT-Plus result came from three WHO regions: Africa, South-East Asia and Western Pacific. The WHO global report 2015 showed that over 80% of new TB cases came from these three regions [5]. We found those regions well represented in our study.

This study recruited a fairly small number of participants (*n* = 134), who may not be representative of other bodies of foreign students in Germany. Caution must be exercised in drawing general conclusions from the results of these studies.

Nevertheless this was the first study describing LTBI prevalence in foreign students in Germany. More studies should be conducted to better determine if and how policies should be developed to screen and test for LTBI in order to achieve better control of the disease.

## Conclusions

In Germany, no specific policies exist for systematic LTBI screening among foreign students in universities and higher education institutions. Considering the steady increase in the proportion of foreigners with active TB compared to the native population and the increasing TB incidence in Germany for the last three years, a policy for LTBI screening and targeted testing among foreign students should be considered to prevent a further increase in TB incidence caused by LTBI reactivation. Our data implies that systematic LTBI testing should be preceded by a questionnaire with a view to testing only those students with risk factors (e.g. previous contact with TB or being born in a high-incidence country).

Our secondary assessment showed that the agreement between the new QFT-Plus and the QFT-GIT was good, which suggests that QFT-Plus might be used in the same format as its predecessor.

## Abbreviations

95% CI: 95% Confidence interval
ACHA: American College Health Association
BCG: Bacille Calmette-Guérin
BGW: Institute for Statutory Accident Insurance and Prevention in the Health and Welfare Services
CVcare: Center of Excellence for Health Services Research in Nursing, Hamburg, Germany
ELISA: Enzyme-linked Immunosorbent Assay
IFN- γ: Interferon-gamma
IGRA: Interferon-gamma release assay
LIST: Lübecker International Student Tutorium
LTBI: Latent tuberculosis infection
OR: Odds ratio
QFT- Plus: QuantiFERON-TB Gold Plus
QFT-GIT: QuantiFERON-TB Gold In-Tube
SD: Standard deviation
TB: Tuberculosis
TST: Tuberculin skin test
U.S.: United States of America
UKE: University Medical Center Hamburg-Eppendorf
WHO: World Health Organization
